# Contextualization of Religion and Entrepreneurial Performance: A Lens of Buddhist Small Business Entrepreneurs

**DOI:** 10.3389/fpsyg.2022.846082

**Published:** 2022-04-13

**Authors:** Lufina Mahadewi, Surachman Surachman, Djumilah Hadiwidjojo, Nur Khusniyah Indrawati

**Affiliations:** Department of Management, Faculty of Economics and Business, University of Brawijaya, Malang, Indonesia

**Keywords:** Buddhist values, entrepreneurial performance, social responsibility, religious values, norms

## Abstract

This study explores the manifestation of Buddhism's conception in underlying entrepreneurial performance. The study is a qualitative research approach with a development direction that comes from successful Buddhist small business entrepreneurs in Bekasi, Indonesia. The interpretive paradigm is used to interpret social life in the reality of successful Buddhist small business entrepreneurs on entrepreneurial performance. Data collection using in-depth interviews with Buddhist small business entrepreneurs in an open-ended format. Data analysis was done in many stages, including domain analysis, taxonomy analysis, component analysis, and theme analysis. The findings indicate that religion acts as an institution that legitimizes the formation of entrepreneurial performance. The performance of Buddhist small business entrepreneurs is manifest in their management of economic or material achievements, and their religious observance in a broad socio-economic context in the relationship of three aspects of human life, namely the individual, social, and environmental, as a form of entrepreneurial practice based on Buddhist values. This research reveals the embodiment of social responsibility for small business Buddhist entrepreneurs which is reflected in entrepreneurial performance through the manifestation of religious values. The findings provide theoretical relevance in institutional theory.

## Introduction

Entrepreneurial performance is closely related to religious values. The linkages that lead to the application of religious beliefs and practices in business have benefits that shape organizational culture and identity (Ravasi and Schultz, 2006; Smith et al., 2019). Entrepreneurial performance is generally measured using objective financial indicators which include profit, business turnover, and position in the market, but in its development, there are performance measures from the personal or subjective side such as business autonomy, self-satisfaction and personal development, and achievement in terms of consumer satisfaction and family welfare (Cooper et al., 1994; Kuratko et al., 1997; Walker and Brown, 2004; Reijonen and Komppula, 2007; Van Praag and Versloot, 2007). Entrepreneurial performance indicators resting on personal or subjective values is posited based on the argument that subjective actions are predictors that are better able to explain business behavior and decision making when compared to objective measurement indicators (Reijonen and Komppula, 2007; Wach et al., 2016; Dijkhuizen et al., 2018). Subjective actions taken by entrepreneurs are often colored by cultural values, one of which is religious values as elements of culture. The influence of religious factors on entrepreneurship is an especially poorly understood phenomenon because the relationship is complex and indirect, our current theoretical perspectives are very rudimentary, and empirical data are scarce.

This research stems from the phenomenon of the success of Buddhist small business entrepreneurs in Bekasi, Indonesia. The result of initial interviews with successful small-scale Buddhist entrepreneurs reveals the phenomenon of the Buddhist perspective on the realization of true happiness and the middle way of harmony and equanimity coloring the perception of entrepreneurial performance in the context of the benefits on a personal basis. This phenomenon provides the motivation for this research to reveal the role of Buddhism in the performance construct of Buddhist small business entrepreneurs. The essence of the small-scale Buddhist entrepreneurs' experience reveals the acculturation of religion and its values in the formation of entrepreneurial character and practice, especially in entrepreneurial performance. This study describes the efforts of Buddhist small-scale entrepreneurs in balancing the application of Buddhism and its values with the fulfillment of entrepreneurial needs in entrepreneurial performance manifestation with the aim of establishing ethical entrepreneurship and achieving prosperity.

Balog et al. (2014) in a literature study of thirty empirical studies in the fields of religion, spirituality, and entrepreneurship, showed a rich relationship between religious values, spirituality in life, and business success. Studies on religious aspects have not been touched in-depth and especially in relation to the role of religious and spiritual values that underlie the area of performance measurement (Balog et al., 2014). Further research is needed that does not focus on measuring entrepreneurial performance in traditional finance but on measuring benefits based on personal or subjective accomplishments such as giving back to society, human resource development, job creation, and achievement of spiritual goals (Joyner et al., 2002; Cornwall and Naughton, 2003; Bakke, 2005; Balog et al., 2014).

In the development of entrepreneurship studies, there are limitations and a lack of development of studies that explore the meaning of entrepreneurial performance in the context of entrepreneurial success based on personal or subjective values (Sen and Oruç, 2009; Baron and Henry, 2011; Gorgievski et al., 2011; Fisher et al., 2014; Gorgievski and Stephan, 2016). The existence of gaps in the design of studies that reveal and explore entrepreneurial performance in the personal perceptions of business actors became the basis for this research. The purpose of this study is to explore the role of Buddhist values in underlying entrepreneurial performance in the context of entrepreneurial success within the scope of subjective personal perception as a form of understanding and assessment of individual business actors on the achievement of business performance.

This study is intended to understand and uncover the multifactorial construct of Buddhist small business entrepreneurs' entrepreneurial performance in the context of personal business success or in the subjective perception of entrepreneurship. The construct concept of entrepreneurial success reflects the criteria for business success perceived by Buddhist small business entrepreneurs. The central issue is the degree to which workplace spirituality might enhance small business performance, as organizational cultures that evidenced higher levels of workplace spirituality were expected to exert a positive effect on employee motivation and commitment. Individuals practicing spiritual leadership at the personal level will experience greater psychological wellbeing, life satisfaction, and physical health: they will also develop a sense of a meaningful and purposeful life and an ability to follow their inner convictions that culminate in a state of self-realization, in alignment with the cultivation of good-quality interpersonal relations. Exploration of the meaning of entrepreneurial performance in the context of personal business success through a qualitative approach is expected to provide a more holistic and integrative representation of the meaning of successful entrepreneurial performance based on the perspective of small business Buddhist entrepreneurs. A qualitative research approach is used to reveal a comprehensive conceptualization of entrepreneurial performance based on understanding and thinking about the meaning of the successful performance of small business Buddhist entrepreneurs.

## Literature Review

The learning process facilitates the entrepreneur's role as a “bicultural mediator” (Peñaloza and Gilly, 1999; Garber, 2001) who supports market diversity and transcultural consumption (Voss et al., 2003) and draws on the contrasting elements of diverse cultural representations (Jamal, 2005). Therefore, acculturation and learning based on experience are interrelated processes in forming business actors. These studies strengthen the basis of this research, which reveals the role of socio-culture, namely religion and its values, in providing a form of social identity that underlies the attitude and personality of an entrepreneur and their entrepreneurial behavior. Religiosity is generally associated with higher ethical attitudes related to corporate social responsibility (Angelidis and Ibrahim, 2004) and the environment (Wolkomir et al., 1997). Brimble et al. (2013) described little difference between the attitudes of more religious and non-religious groups in investing in socially responsible activities. The study found that both of the two groups grouped financial criteria as more important than social investment criteria, which were influenced by different religious philosophies. Religion is believed to have a positive and significant influence on different levels of entrepreneurial activity (Zelekha et al., 2014). Angelidis and Ibrahim (2004) noted a significant relationship between the level of religion and attitudes toward the economic and ethical components of corporate social responsibility. Brammer et al. (2007) argued that religion in the context of individuals does not place different preferences for corporate responsibility but tends to have a broader understanding of business social responsibility compared to individual business actors who do not have religious beliefs.

These studies provide the basis for this study to reveal the role of Buddhism in entrepreneurial performance that underlies the measurement in the context of personal benefits. This study describes the role of religion and its values in the underlying practice of applying the benefits of entrepreneurial performance. This research is motivated by previous studies in which indicators of the success of an entrepreneur are not only measured by traditional financial performance measurements but through indicators of entrepreneurial achievement in providing benefits to community and stakeholder development or the personal goals of the entrepreneur (Joyner et al., 2002; Cornwall and Naughton, 2003; Bakke, 2005).

Ibrahim et al. (1991) indicated a significant and positive relationship between religious values and entrepreneurial performance such as business growth, productivity, and human capital performance. Gill and Mathur (2018) and Li et al. (2019) stated that religious beliefs and gender factors encourage involvement in social responsibility, especially when the entrepreneur is a woman. In addition, religious beliefs also encourage lower borrowing costs. Hassan et al. (2015) revealed that business experience and performance are affected by religion or belief. Baharun and Kamarudin (2001) and Cheung (2004) outlined that religious values do not affect the achievement of entrepreneurial performance and spiritual values possessed by entrepreneurs provide a competitive disadvantage, especially in achieving entrepreneurial financial performance. Dilmaghani (2011) showed that religion has a negative relationship and is significant to income.

Ketola et al. (2009), Chowdhury (2018), and Zaman et al. (2018) showed that the intrinsic motivation of religiosity, namely the religious beliefs themselves, play an important role in increasing the responsibility and concern of entrepreneurs for environmental welfare. Religious beliefs shape entrepreneurial behavior in the practice of implementing social responsibility. Wisker et al. (2020) denoted a significant positive relationship between religion and belief and corporate social responsibility. The findings of qualitative data (Wisker et al., 2020) indicated that there is no significant difference in the three religions studied, namely Christianity, Islam, and Hinduism, in showing the role of religiosity in the performance of social responsibility in small and medium enterprises.

Based on these studies, there emerges a research gap on the role of religion in underlying entrepreneurial performance as indicated by various findings. The research gap is also shown in the limited research that explores the point of view of Buddhist entrepreneurs in the context of Buddhism and the perspective of Buddhist Economics. This research gap is used as the basis for reviewing and strengthening the conception of the role of religion and its values in supporting entrepreneurial performance from the perspective of small-scale Buddhist entrepreneurs. The output of this study is expected to provide an embodiment of the role of religion as an instrumental mechanism in encouraging the role of entrepreneurship in shaping the business and economic environment as well as its identity as an entrepreneur.

## Research Methods

This study uses a phenomenological research tradition with an interpretive paradigm. This paradigm is used to understand and describe social events that originate from interaction by focusing on understanding and interpreting language in the reality of a successful Buddhist small business entrepreneurs in Bekasi, Indonesia. The information in this study was collected by using interviews. An interview guide in the form of a question guide was prepared and used in the interview process as a reference. Although during the interview process, an intuitive approach was also used to ensure that the unique experience of each informant could be illustrated. Each interview was recorded by audio.

The in-depth interview process was carried out to dig up intentional information through the process of informants telling their experiences profoundly. Interviews were conducted in a semi-structured and flexible manner according to the situation in the field. The number of informants needed is based on the saturated sample criteria. The informants are 14 Buddhist small business entrepreneurs who are successful and have experience consciously manifesting the practice of Buddhist values in the context of their everyday reality and entrepreneurial performance, and seize the ability to express a comprehensive picture of their experiences. The interview question guide was prepared using an open-ended format to encourage small business Buddhist entrepreneurs to openly reveal the experience of manifesting Buddhist values in their entrepreneurial performance. Interviews were recorded and tested for the validity of the data by testing credibility, transferability, dependability, and confirmability. The research output produces repeated implied and explicit themes that form propositions of the role of religion and its values in the conception of Buddhist Economics in underlying entrepreneurial performance on the basis of personal values. Horizontal mapping is used (Creswell, 2007) to ensure that there is no repetition and redundancy of important statements. Abstraction of meaning is compiled based on important statements sourced from interview excerpts of each informant. The choice of statements from each informant will be converted into meaningful meanings.

The stages of data analysis comprise the domain, taxonomy analysis, component analysis, and theme analysis (Spradley, 1980). Domain analysis is used to examine the overall picture of the role of Buddhist values in underlying entrepreneurial performance. Domain analysis presents the semantic relationship of the role of Buddhist values in underlying entrepreneurial performance. The taxonomic analysis is carried out to conduct an in-depth search of the domains that have been arranged based on the selected domains. In the componential analysis, procedures are carried out to construct contrast in the domain obtained through interview data, observation, and field documentation. Theme analysis was conducted to determine implied and explicit themes that were repeated in a number of domains and acted as a liaison. Theme analysis is used to explore linkages that combine cross-domains consisting of a series of domain, taxonomy, and componential analyses as well as narrative descriptions that compose a theme architecture. The themes for all the informants were examined and similarities emerged across the informants resulting in the representation of the final main theme from all the informants. The theme analysis presents themes on the role of Buddhism that underlie the entrepreneurial performance of small business Buddhist entrepreneurs.

## Results

The findings of this study reveal and provide an understanding of the role of Buddhism in exploring and interpreting the meaning of entrepreneurial performance in the context of personal business success. There is a similar understanding of each essence of experience conveyed by the informants regarding the role of Buddhist values in underlying entrepreneurial performance, namely *Sabbe Satta Bhavantu Sukhitattā*, Balance, Karma, *Anana Sukha*, Use of wealth (mental attitude toward wealth), and the Noble Eightfold Path. The themes revealed in this study have implications as a form of measuring entrepreneurial success on a personal basis and the values that underlie the performance of Buddhist small business entrepreneurs. Entrepreneurial performance emphasizes achieving social welfare, moderating the implementation of entrepreneurship wisely, and maximizing the use of internal entrepreneurial results.

### Social Welfare

Buddhist values have a positive impact on the formation of trust both in interpersonal relationships and at the organizational level (Vu and Tran, 2019). Buddhist small business entrepreneurs interpret the Buddhist perspective of *Sabbe Satta Bhavantu Sukhitattā* through the application of appropriate entrepreneurial practices for the attainment of self-happiness by not harming other beings with the application of virtue. The concept of achieving entrepreneurial performance implies that the material achievements obtained from entrepreneurial performance provide benefits for social welfare, not an obsession with oneself that causes suffering. The economic model in Buddhism provides meaning to small business Buddhist entrepreneurs that the achievement of entrepreneurship is not solely directed at the maximum accumulation of wealth, but also pays attention to the process or method of obtaining and utilizing it. Buddhism ensures that wealth leads to the development of a potential quality of life (Payutto and Evans, 1994).

The perspective of Sabbe Satta Bhavantu Sukhitatta encourages the direction of entrepreneurial performance in harmonization with the causal process and three interrelated aspects of human life. Buddhist small business entrepreneur Po Seng stated the following:

“*Success is when it can be useful for others. I do not want to harm others, it is better for me to lose than to harm others. As long as there is an opportunity for me to do good, I always try to do good. I try to make all sentient beings happy.” (Po Seng)*.

In Buddhism, namely the Karaniyametta Sutta or Metta Sutta, the expression *Sabbe Sattā Bhavantu Sukhitattā* owns the meaning of developing loving-kindness *or Metta* for all living beings (Hansen, 2008). The underlying assumption is a sustained determination or desire to reduce suffering (Lama and Thupten, 1995; Vu and Tran, 2019). The practical implication that emerges is an appreciation of the diversity of individual and contextual cultural differences that foster an inclusive entrepreneurial climate for spiritual wellbeing in organizations (Nishii and Rich, 2014; Gotsis and Grimani, 2017; Vu and Tran, 2019). Buddhist small business entrepreneurs interpret *Sabbe Satta Bhavantu Sukhitattā* as a manifestation of efforts to encourage the formation of stakeholders' confidence in entrepreneurship through the efforts shown to the concern for their welfare in three aspects of life.

The essence of the experience of Buddhist small business entrepreneurs illustrates that entrepreneurial performance is a form of approach that aims to direct entrepreneurial performance managerially to build stakeholder trust. Its application is carried out through the realization of an attitude of wisdom with an emphasis on considerations of moral and ethical weight. *Sabbe Satta Bhavantu Sukhitattā's* conception provides direction and goals to achieve entrepreneurial performance on how entrepreneurship proceeds in harmony and supports three aspects of life. The achievement of the entrepreneurial performance of Buddhist small business entrepreneurs is based on the application of the personal values of Buddhist entrepreneurs that are in line with the Buddhist perspective, namely compassion, honesty, and simplicity. Entrepreneurial performance is not directed at the satisfaction of personal desires related to material achievement. The selflessness and attachment of the Buddhist perspective provide implications on the important role of ethical values and considerations in the overall causal process in all three aspects of life in every entrepreneurial strategic decision that impacts entrepreneurial performance. The harmonization of the three aspects of life in the Buddhist perspective forms a system of ethical judgment or judgment on mental conditions and causal relationships of every strategic decision that contributes to entrepreneurial performance.

The essence of the Buddhist small business entrepreneurs' experience reveals that karma plays a role in underlying the achievement of entrepreneurial performance. The karmic perspective is used as an indicator or personal value that underlies entrepreneurial performance. Subjective actions based on the personal values of entrepreneurs shape economic behavior and entrepreneurial decision-making (Reijonen and Komppula, 2007; Wach et al., 2016; Dijkhuizen et al., 2018). Karma provides a contextual managerial approach to the formation of an ethical and sustainable entrepreneurial culture through the internalization of the incorporation of non-self and impermanent concepts that form the implementation of ethical principles, equality, and honesty.

Entrepreneurial performance targets are manifested in strategic and operational decisions aimed at improving welfare for three aspects of life. Entrepreneurial performance does not focus on material achievement at the expense of general welfare which has karmic consequences. In Buddhist cosmology, the form of the rebirth of an individual is determined by karma (Hansen, 2008). Therefore, the meaning of achieving entrepreneurial performance is articulated by Buddhist small business entrepreneurs as experience in answering entrepreneurial dilemmas. Contrariety to integrate the pursuit of financial goals with the application of entrepreneurial goals as an expression of practicing religious values. Religion configures the role of socio-culture as an indicator in the contextual aspect of entrepreneurship. Religion acts as an environmental moderator and perspective lens in the strategic management of companies (Valliere, 2008). The view of Karma in Buddhism is not only an abstract aspect. Karma internalizes its meaning in the manifestation of entrepreneurial performance achievement in balancing the conflicting strategic objectives of entrepreneurship from the financial objective side and the formation of ethical entrepreneurship for general welfare.

### Moderation at the Optimal Limit of Entrepreneurship

Buddhist small business entrepreneurs interpret balance in entrepreneurial performance which focuses on achieving prosperity that leads to wise consumption. Entrepreneurial performance is based on Buddhist values of balance, simplicity, and honesty. Entrepreneurial performance is defined by moderation at the optimal limit of business activity implementation. Buddhist small business entrepreneur Suwandi stated:

“*Business success for me if the business is able to grow financially but the most important thing is inner satisfaction. When I have loyal customers and do not turn away because of the quality of the product and my ability to respond to customer needs is also a successful entrepreneurial performance for me. Success also means knowing your limits and not being tormented in making money and running a business. The value of Buddhism which teaches to live as it is, avoid risks and realize that life has a cause and effect relationship. That's the value that I hold in my business because kindness is a religious teaching, so the Buddhist values that I hold in running a business are living a simple life, not forcing one's own strength, living a grateful and happy life.” [sic] (Suwandi)*.

With a balanced perspective, Buddhist small business entrepreneurs seize an internal direction and ability to recognize entrepreneurial desires or ambitions that lead to suffering. Through the principles of the *Middle Way of Buddhism*, small business Buddhist entrepreneurs reflectively direct entrepreneurial attainment to a life that is balanced with material pursuits through ethical considerations and beneficence in the three aspects of life. Small business Buddhist entrepreneurs focus on the long-term orientation of achieving entrepreneurial performance for quality of life. The self-reflection that emerges from the internalization of the balanced perspective provides direction for small business Buddhist entrepreneurs to consider the impact of entrepreneurial decisions on long-term entrepreneurial performance.

Buddhist small business entrepreneurs render priority to long-term oriented strategic entrepreneurial decisions with a tendency to avoid short-term entrepreneurial decisions that are oriented toward material pursuits, by full careful consideration of cause and effect also ethical-moral weight. The perspective of balance or the Middle Way reflects the character of small business Buddhist entrepreneurs' decision-making which is based on a sense of ownership and responsibility for the inherent consequences of each entrepreneurial decision.

By applying the Buddhist conception of *Right Livelihood*, Buddhist small business entrepreneurs prevail in the Buddhist view of economics, which is the achievement of goals for a good personal, social, and environmental life (Payutto and Evans, 1994). The findings of this study provide a construction of the meaning and interpretation of the role of Buddhist conception, namely *Right Livelihood* in individual Buddhist small business entrepreneurs in achieving entrepreneurial performance. This research also enriches previous research on the role of religious beliefs in the context of entrepreneurial decision-making from various religious traditions.

### Utilization of Internal Business Results

The essence of the experience of Buddhist small business entrepreneurs reveals that the Buddhist perspective, namely *Anana Sukha* or the happiness of being free from debt, plays a role in achieving entrepreneurial performance. *Anana Sukha* is a form of happiness for lay householders (Hansen, 2008).

The narrative of the experience of Buddhist small business entrepreneurs reveals the meaning of achieving entrepreneurial performance with the happiness of being free from debt. Buddhist small business entrepreneurs are in a position that can harness the material achievements resulting from entrepreneurial performance. In the *Digha Nikaya*, happiness lies in the economic foundation (Obadia, 2011) by living in economic security (*atthi-sukha*), enjoying wealth (*bhoga-sukha*), and being free from debt (*anana-sukha*). *Anana Sukha*'s perspective is interpreted as a form of freedom from the burden of suffering and self-happiness which is manifested in the measurement of entrepreneurial performance. The interpretation of the manifestation of religious values provides an understanding of the magnitude of the role of religion in entrepreneurship. The Buddhist small business entrepreneur Ferry stated the following:

“*Success in entrepreneurship for me if I am able to meet the needs of life without being in debt. I don't want to have a nice and luxurious house from the results of my business but have a business debt to the bank.” (Ferry)*.

Success in entrepreneurship does not focus on maximizing the financial side but on the acquisition of wealth that is ethical, not detrimental, and able to use internal business results without having the burden of debt. Understanding the concept of acquiring wealth forms an understanding of the implementation of entrepreneurship without any elements of exploitation, ethics and does not become a burden that disturbs the balance in other aspects of life. Small business Buddhist entrepreneurs use internal business results appropriately for entrepreneurial development without any ties to external funding. Buddhism focuses on this relationship through the understanding that benefiting and sharing with others can only be done with the wealth gained (Deegalle, 2016), while Buddhism is not against any form of attainment of wealth.

The achievement of entrepreneurial performance is also interpreted by small business Buddhist entrepreneurs according to the perspective of using wealth for self and family life, community harmony, and supporting good deeds. One form of good practice carried out by Buddhist small entrepreneurs is entrepreneurial performance in meeting employee welfare. Employees are treated as co-workers. For employees who are loyal and do good work, Buddhist small business entrepreneurs treat them as part of the family and have a sense of responsibility for their wellbeing. Employee welfare efforts are demonstrated by providing knowledge and expertise for the purpose of self-reliance.

## Discussions

Entrepreneurial performance is interpreted by Buddhist small business entrepreneurs as a form of entrepreneurial value. The entrepreneurial performance of Buddhist small business entrepreneurs is described in the performance that does not focus on material achievement but the use of entrepreneurial financial performance on the realization of harmony and prosperity for three aspects of life. Successful entrepreneurial performance for small business Buddhist entrepreneurs is also reflected as the ability to use the results of their performance for ethical use in three aspects of life without the debt that causes burden or suffering.

This study addresses the limitations of the entrepreneurial literature that focuses on traditional performance measurement in financial measures (Balog et al., 2014). This study supports the inevitability of institutional theory by providing a successful construct of entrepreneurial performance in the context of the perception or personal value base of small business Buddhist entrepreneurs.

This study reveals that small business Buddhist entrepreneurs believe that the achievement of entrepreneurial performance from a financial perspective is the result of achieving entrepreneurial practices that are carried out in accordance with religious values and become a resource for achieving the true meaning of entrepreneurial performance. This study also shows a strong interrelation between religious values in the life of Buddhist small business entrepreneurs and successful entrepreneurial performance. The performance of Buddhist small business entrepreneurs is manifested in the form of management performance in the forms material and immaterial, which is reflected in economic or material achievements, religious observance, and in a broad socio-economic context in the relationship of three aspects of human life, namely individual, social, and environmental, which is the impact of entrepreneurial practices based on Buddhist values.

The entrepreneurial performance of small-scale Buddhist entrepreneurs is a manifestation of social responsibility through the practice of Buddhist values in the welfare of three aspects of life. The embodiment of social responsibility illustrates the form of entrepreneurial legitimacy in its relevance to institutional theory.

Institutional theory explains how organizations manifest from an institution through beliefs, rules, and norms as a means of legitimacy (Selznick, 1984) and value systems that shape legitimacy. Institutional theory provides relevance to this research phenomenon of institutionalization of entrepreneurship based on culture, namely religion and its value in shaping business legitimacy. This theory is used to reinforce the conception of the role of religion and its values in underlying entrepreneurial performance on the basis of values or personal measurements such as achievement of stakeholders. This theory suggests the relevance of the findings of this study that entrepreneurial performance is a form of social responsibility for small business Buddhist entrepreneurs in fulfilling responsibilities in three aspects of life. This theory also reveals the relevance of the realization of the form of social responsibility of Buddhist small business entrepreneurs which is reflected in entrepreneurial performance through the manifestation of religious values. Field data found that the values of Buddhism underlie the performance measurement of Buddhist small business entrepreneurs. This study shows the relevance between the findings of field data and institutional theory.

Based on the essence of the experience of Buddhist small business entrepreneurs, research propositions can be drawn up that describe the value of Buddhism when internalized into entrepreneurial performance.

Proposition 1: *Sabbe Satta Bhavantu Sukhitatta* conveys the meaning of the entrepreneurial performance of Buddhist small business entrepreneurs who do not focus on material achievement but on the utilization of entrepreneurial financial performance to realize the harmonization and welfare of three aspects of life, namely the individual self, society, and the environment.
  *Sabbe Satta Bhavantu Sukhitatta* provides an entrepreneurial performance perspective, namely the implementation of entrepreneurial practices that regard the suitability of ethical and moral values and do not cause adverse impacts on self-interest and society.
Proposition 2: The Buddhist perspective of Balance underlies Buddhist small business entrepreneurs in reflecting the achievement of entrepreneurial performance through ethical considerations and the usefulness of entrepreneurship to moderate the achievement of balance between the goals of material pursuits and harmonization in three aspects of life. The perspective of balance in Buddhism underlies the strategic decision orientation of entrepreneurship that focuses on wellbeing and balance in life.
Proposition 3: The Buddhist perspective of *Karma* underlies Buddhist small business entrepreneurs to reflect on the causes and effects that arise from entrepreneurial strategic decisions and goal setting. Karmic reflexivity underlies the balance of achievement of entrepreneurial performance in terms of objective financial achievement and the application of ethical entrepreneurial practices for the benefit of the general welfare to create good karma for the quality of life.
Proposition 4: *Anana Sukha* underlies the meaning of entrepreneurial performance of Buddhist small business entrepreneurs on achieving entrepreneurial performance that does not cause burdens and conditions of dependence on debt. *Anana Sukha* emerges meaning to the success of entrepreneurial performance on utilizing internal business results without debt in carrying out ethical entrepreneurial practices and providing benefits to other aspects of life.
Proposition 5: The use of wealth (*mental attitude toward wealth*) in the Buddhist perspective implies that entrepreneurial financial achievement is a resource for creating benefits for three aspects of life in the form of achieving general welfare and developing the quality of life of Buddhist small business entrepreneurs.
Proposition 5.31: *The Noble Eightfold Path* (*Right Livelihood*) in the perspective of Buddhism underlies the meaning of achieving entrepreneurial performance in Buddhist small business entrepreneurs, namely forming and practicing entrepreneurship by obtaining material or wealth achievements in accordance with the conception of *Right Livelihood* to realize the attainment of prosperity and harmony in balance.

**Figure 1:**
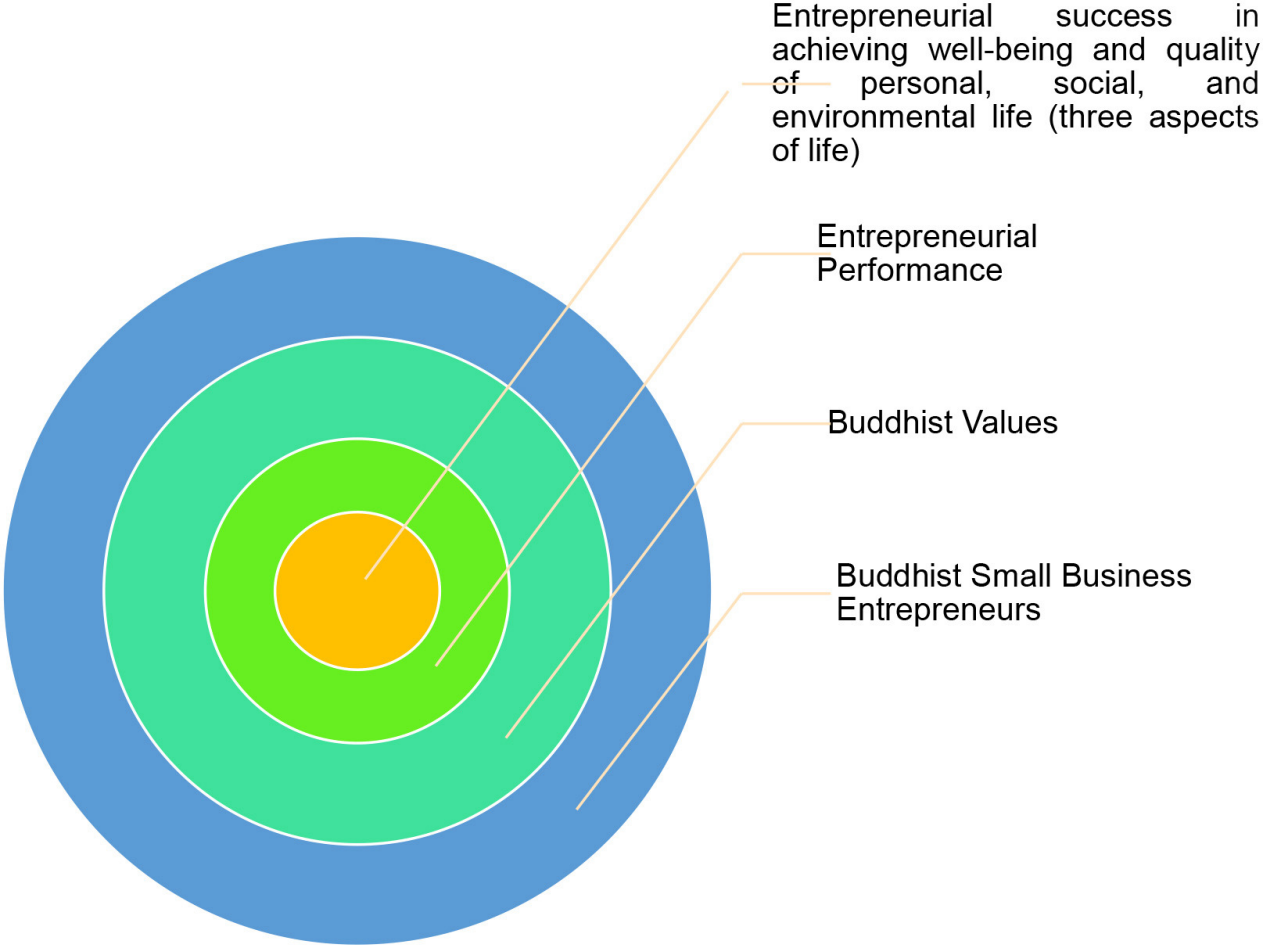
The role of Buddhism in entrepreneurial performance. Source: Lufina Mahadewi.

Based on a partial and holistic study of the theme of the role of Buddhism in entrepreneurial performance, a model for the role of Buddhism in underlying entrepreneurial performance is developed. The model framework is visualized in Figure 1.

Figure 1 shows values or conceptions of Buddhism that underlie entrepreneurial performance to realize true happiness according to the Buddhist perspective, namely achieving prosperity and developing the quality of life in three aspects of life (personal, social, and environmental). Buddhism is embodied in the meaning of successful entrepreneurial performance of Buddhist small business entrepreneurs through the enlightenment of religiosity by understanding the desire and stimulant power in entrepreneurial economic activities.

## Conclusions and Recommendations

The findings of this study reveal the construction of a contingency framework embodied by Buddhist small business entrepreneurs in the perspective of entrepreneurial performance. Religion manifests its institutionalization process through normative, cognitive, and regulatory aspects in institutional activities that occur because of the actualized role of Buddhist small business entrepreneurs. The actualization is realized by Buddhist small business entrepreneurs in the perspective of entrepreneurial performance which illustrates that religion shapes the social context through its role at the individual level. Small business Buddhist entrepreneurs embody the construction of their entrepreneurial performance through the general cultural-institutional arguments approach. The findings of this study can be taken into consideration in making local government policies in formulating policies for fostering small-scale entrepreneurship by observing the harmony of religious values and norms. The limitation of this study is that it only discusses the issue from the angle of one religious value; further research can combine inter-religious values to observe the phenomenon of Buddhist small business entrepreneurs from the wider perspective of entrepreneurial performance.

## Data Availability Statement

The raw data supporting the conclusions of this article will be made available by the authors, without undue reservation.

## Author Contributions

LM conceived of the presented idea. SS and LM developed the theory and performed the computations. DH verified the analytical methods. NI encouraged LM to investigate topic and supervised the findings of this work. All authors discussed the results and contributed to the final manuscript.

## Conflict of Interest

The authors declare that the research was conducted in the absence of any commercial or financial relationships that could be construed as a potential conflict of interest.

## Publisher's Note

All claims expressed in this article are solely those of the authors and do not necessarily represent those of their affiliated organizations, or those of the publisher, the editors and the reviewers. Any product that may be evaluated in this article, or claim that may be made by its manufacturer, is not guaranteed or endorsed by the publisher.
